# A simplified frailty index and nomogram to predict the postoperative complications and survival in older patients with upper urinary tract urothelial carcinoma

**DOI:** 10.3389/fonc.2023.1187677

**Published:** 2023-10-11

**Authors:** Jianyong Liu, Haoran Wang, Pengjie Wu, Jiawen Wang, Jianye Wang, Huimin Hou, Jianlong Wang, Yaoguang Zhang

**Affiliations:** ^1^ Beijing Hospital, National Center of Gerontology, Institute of the Geriatric Medicine, Chinese Academy of Medical Sciences & Peking Union Medical College, Beijing, China; ^2^ Beijing Hospital Continence Center, Beijing, China

**Keywords:** upper urinary tract urothelial carcinoma, frailty, postoperative complication, prognosis, risk stratification, random forest

## Abstract

**Purpose:**

This study was designed to investigate the clinical value of a simplified five-item frailty index (sFI) for predicting short- and long-term outcomes in older patients with upper urinary tract urothelial carcinoma (UTUC) patients after radical nephroureterectomy (RNU).

**Method:**

This retrospective study included 333 patients (aged ≥65 years) with UTUC. Patients were classified into five groups: 0, 1, 2, 3, and 3+, according to sFI score. The variable importance and minimum depth methods were used to screen for significant variables, and univariable and multivariable logistic regression models applied to investigated the relationships between significant variables and postoperative complications. Survival differences between groups were analyzed using Kaplan-Meier plots and log-rank tests. Cox proportional hazards regression was used to evaluate risk factors associated with overall survival (OS) and cancer-specific survival (CSS). Further, we developed a nomogram based on clinicopathological features and the sFI. The area under the curve (AUC), Harrel’s concordance index (C-index), calibration curve, and decision curve analysis (DCA) were used to evaluate the nomogram.

**Result:**

Of 333 cases identified, 31.2% experienced a Clavien-Dindo grade of 2 or greater complication. Random forest–logistic regression modeling showed that sFI significantly influenced the incidence of postoperative complications in older patients (AUC= 0.756). Compared with patients with low sFI score, those with high sFI scores had significantly lower OS and CSS (p < 0.001). Across all patients, the random survival forest–Cox regression model revealed that sFI score was an independent prognostic factor for OS and CSS, with AUC values of 0.815 and 0.823 for predicting 3-year OS and CSS, respectively. The nomogram developed was clinically valuable and had good ability to discriminate abilities for high-risk patients. Further, we developed a survival risk classification system that divided all patients into high-, moderate-, and low-risk groups based on total nomogram points for each patient.

**Conclusion:**

A simple five-item frailty index may be considered a prognostic factor for the prognosis and postoperative complications of UTUC following RNU. By using this predictive model, clinicians may increase their accuracy in predicting complications and prognosis and improve preoperative decision-making.

## 1 Introduction

Urinary tract urothelial carcinoma (UTUC), a transitional cell carcinoma of the renal pelvis and ureter, is a rare tumor with poor prognosis and an incidence of approximately 2 per 100,000 in developed countries (1–3). Due to lack of symptoms and delayed diagnosis, tumors usually develop muscle invasion or local progression, resulting in a worse prognosis than bladder cancer. For patients with T2/T3 and T4 stage tumors, the 5-year specific survival rates are less than 50% and 10%, respectively (1). Despite advances in systemic therapy and immunotherapy in recent years (4), radical nephroureterectomy (RNU) with bladder cuff resection remains the standard treatment for high-risk UTUC, regardless of tumor location. Factors related to prognosis after RNU mainly include clinicopathological features, such as lymphovascular invasion, pathological TNM stage, concomitant carcinoma in situ, and tumor multifocality, among others (1).

Population aging is among the most important trends worldwide, and the peak incidence of UTUC is in individuals aged 70–90 years, including high prevalence in people with frailty (1, 5). Frailty is a multidimensional physiological syndrome, typically involving loss of reserves (such as energy, physical capacity, cognition, and health) and increased vulnerability (6, 7). Consequently, frail older patients are more likely to have negative surgical outcome in the perioperative period. Frailty before surgery has been shown to objectively predict postoperative complications, mortality, and extended hospital stay (8–16). The Comprehensive Geriatric Assessment (CGA) is the gold standard for determining frailty (17), however, it requires specialist expertise and is time-consuming, making it less thanideal for use in routine clinical practice. Therefore, many scholars have attempted to use alternative methods to measure the degree of frailty. Fried and colleagues first proposed a standardized phenotype of frailty in older adults and demonstrated that frailty is associated with risk of adverse outcomes in such individuals (6). In 2005, the Canadian Study of Health and Aging developed a 70-item Frailty Index based on the presence and severity of current diseases, ability to perform activities of daily living, and physical and neurological signs from clinical examinations (7). In addition, several other indicators have also been shown to accurately assess frailty, such as the modified Frailty Index (mFI) (18–20) and the Memorial Sloan Kettering-Frailty Index (8). Although these indicators measurement indices represent improvements, their feasibility under the pressure of clinical work remains questionable. Therefore, the original modified frailty index (mFI-11) was modified to generate the mFI-5 by removing some variables, and the ability of mFI-5 to characterize frailty has been demonstrated in many studies (9, 21–27). Overall, frailty is an independent predictor of adverse outcomes after surgery, particular in older adults.

While many clinicopathological indicators may predict prognosis and morbidity in patients with UTUC, indicators that are simpler and easier to use in a clinical setting are also needed. Furthermore, preoperative risk and prognosis assessment tools currently used in older patients with UTUC do not include frailty. Therefore, in this study, we aimed to assess the prognostic significance of a simplified five-item frailty index on short-term (postoperative complications) and long-term (overall survival (OS) and cancer-specific survival (CSS)) outcomes after RNU for elderly patients with UTUC.

## 2 Patients and methods

### 2.1 Study population

Patients aged > 65 years undergoing RNU in the Department of Urology, Beijing Hospital, National Center of Gerontology, were retrospectively enrolled. The inclusion criteria were as follows: (1) patients aged ≥65 years; (2) patients undergoing RNU; (3) patients with histopathologically verified urothelial carcinoma; (4) patients without metastatic lesions; (5) patients with complete clinical and follow-up information. A total of, 333 elderly patients with UTUC (age ≥ 65 years) were included in the final study.

### 2.2 Data collection and definition of variables

Preoperative patient demographic and general clinical data were collected from patient electronic hospital records, including patient age, weight, height, body mass index (BMI), urine pathology, and presence of preoperative hydronephrosis. Patient comorbidities, including smoking status in the last year, chronic obstructive pulmonary disease, congestive cardiac failure, anti-hypertensive treatment, preoperative acute renal failure, diabetes mellitus, and functional status, were recorded. Pathological findings included cancer-related data, such as tumor stage, tumor grade, tumor site, tumor side, tumor size, lympho-vascular invasion (LVI), multifocality, concomitant CIS (carcinoma in situ), and surgical margin. Intraoperative and postoperative data, including American Society of Anesthesiologists (ASA) score, surgical treatment, blood transfusion, operation time, and postoperative complications, were obtained from anesthesia records, operative documents, and electronic medical record systems.

The simplified five-item frailty index (sFI) was calculated using five dichotomous comorbidity categories, as follows: history of diabetes mellitus, history of congestive heart failure, hypertension requiring medication, history of chronic obstructive pulmonary disease, and functional status, as previously reported (21–23, 28, 29). Functional status was defined as requiring assistance for any activities of daily living (ADLs), including bathing, feeding, dressing, and mobility. Each comorbidity was assigned one point, giving scores in the range,0 to 5. As a result of limitations in sample size, patients were divided into five groups based on sFI score, as follows: 0, 1, 2, 3, and 3+. In this study, OS was defined as the length of time from diagnosis to death or last follow-up visit and. CSS was calculated from the date of surgery to the date of death from cancer. Postoperative complications were graded according to the Clavien-Dindo classification system.

### 2.3 Features selection

Rapid advances in artificial intelligence have led to an explosion in the use of machine learning to develop prediction models for various diseases in recent years (30–33). Random forest is a classification algorithm comprising numerous many decision trees. Thus, random forests can better predict outcomes than individual classification trees, which can also automatically identify nonlinear effects between variables. A random survival forest model can be constructed by combining random forest and traditional survival analyses; random survival forest models are susceptible to outliers and should, therefore, be used in conjunction with, rather than as a complete replacement for, traditional survival analysis.Thus, we first collected data on clinical and pathological parameters. Then, variable screening was conducted using two methods, variable importance (VIMP) and minimal depth. A VIMP value less than 0 indicates that a variable reduces prediction accuracy, while a value greater than 0 indicates that a variable improves prediction accuracy. The minimum depth rule determines the importance of each variable to the final event by calculating the minimum depth when running to the last node; where variables with smaller values are more critical to the model. We use random forest’s VIMP and minimal depth method to select variables for predictive model construction.

### 2.4 Statistical analysis

Statistical analyses were performed using R4.2.1 (R Core Team, Vienna, Austria). The Chi-square or Fisher’s exact tests were used to compare categorical variables. Continuous variables are described using mean with standard deviation (SD) and medians with interquartile range (IQR). The Kaplan–Meier method was applied to evaluate OS and CSS rates, and the log-rank test used to analyze significance. Univariable and multivariable Cox regression models were applied to assess risk factors associated with OS and CSS. Similarly, a multivariable logistic regression model was applied to assess relationships between variables and postoperative complications. All significant factors were incorporated into anomogram. The concordance index (C-index) and the area under the time-dependent receiver operating characteristic (ROC) curve (time-dependent AUC) were used to assess the discriminative ability of models. Calibration ability was evaluated using calibration plots. Decision Curve Analysis (DCA) was used to determine the clinical usefulness of the nomogram by calculating the net benefit at different threshold probabilities. Patients were divided into three groups according to total nomogram scores: low-, intermediate-, and high-risk. P<0.05 was considered statistically significant.

## 3 Results

### 3.1 Patient characteristics

The clinical characteristics of the 333 patients included in this study are shown in **Table 1**. Median follow-up time was 36.67 months (IQR: 21.83–67.93 months). Mean patients age was 74 ± 5.80 years, and mean operative time was 227 ± 71.6 minutes. Of patients, 264 were *<* 80 years old, and 69 were ≥ 80 years of age. Further, 148 (44.4%) patients were male, and 185 (55.6%) were female. Median BMI was 24.14 kg/m^2^ (IQR: 21.64–26.40 kg/m^2^). Pathological tumor stage was pT1, T2, T3, and T4 in 82 (24.6%), 103(30.9%), 132 (39.6%), and 16 (4.80%)patients, respectively. Primary tumors located in the renal pelvis accounted for 39.3%, while 60.7% were in the ureter. Approximately 12.6% of patients had multiple tumors at diagnosis, and the majority of patients (82.0%) had a high histologic grade. Patients were grouped according to sFI scores, as follows: sFI 0, 31 (9.31%); sFI 1, 87 (26.1%); sFI 2, 86 (25.8%); sFI 3, 88 (26.4%); and sFI 3+, 41 (12.3%). Within this population, 21 (6.31%) patients had a history of cerebral infarction, and 45 (13.5%) of renal failure.

**Table 1:** Clinical characteristics of study population.

Characteristics	Number (Percentage)
Gender	
Male	148 (44.4%)
Female	185 (55.6%)
Age (years)	
<80	264 (79.3%)
≥80	69 (20.7%)
BMI (kg/m^2)^	
<25	197 (59.2%)
≥25	136 (40.8%)
Side	
Left	184 (55.3%)
Right	149 (44.7%)
Site	
Pelvis	131 (39.3%)
Ureter	202 (60.7%)
Approach	
Laparoscopic	151 (45.3%)
Open	182 (54.7%)
Ureteroscopy	
No	279 (83.8%)
Yes	54 (16.2%)
Hematuresis	
No	88 (26.4%)
Yes	245 (63.6%)
Urine pathology	
No	79 (23.7%)
Yes	254 (76.3%)
Symptom	
No	59 (17.7%)
Yes	274 (82.3%)
Hydronephrosis	
No	72 (21.6%)
Yes	261 (78.4%)
Multifocality	
No	291 (87.4%)
Yes	42 (12.6%)
Size (cm)	
<5	302 (90.7%)
≥5	31 (9.31%)
LVI	
No	280 (84.1%)
Yes	53 (15.9%)
Tis	
No	318 (95.5%)
Yes	15 (4.50%)
T stage	
T1	82 (24.6%)
T2	103 (30.9%)
T3	132 (39.6%)
T4	16 (4.80%)
Margin	
Negative	321 (96.4%)
Positive	12 (3.60%)
pN+	
N0&Nx	311 (93.4%)
N+	22 (6.61%)
Grade	
Low	60 (18.0%)
High	273 (82.0%)
History of previous abdomen surgery	
No	245 (73.6%)
Yes	88 (26.4%)
History of smoking	
No	265 (79.6%)
Yes	68 (20.4%)
Blood transfusion	
No	264 (79.3%)
Yes	69 (20.7%)
Surgery time(hours)	3.78 ± 1.19
sFI	
0	31 (9.31%)
1	87 (26.1%)
2	86 (25.8%)
3	88 (26.4%)
3+	41 (12.3%)
Length of Stay (days) (Mean ± SD)	11.5 ± 6.08
History of cerebral infarction	
No	312 (93.7%)
Yes	21 (6.31%)
Renal failure	
No	288 (86.5%)
Yes	45 (13.5%)

BMI, body mass index; CIS, carcinoma in situ; LVI, lympho-vascular invasion.

### 3.2 Postoperative complications

At least one Clavien-Dindo postoperative complication grade ≥ 2 developed in 104 (31.2%) patients (**Table 1** **)**. A breakdown of postoperative complications is presented in **Table 2**. A total of 138 postoperative complications were observed in this study: Clavien-Dindo grade 2, 95 (68.8%); Clavien-Dindo grade 3, 10 (7.2%); Clavien-Dindo grade 4, 31 (22.5%); and Clavien-Dindo grade 5, 2 (1.4%). The number of postoperative complications differs from the number of patients who experienced them, as some patients experienced more than one complication. Delirium was the most common complication (n=11, 9.6%), followed by Incision fat liquefaction (defined as necrosis of adipose tissue without infection) (n=11, 8.0%), requiring intensive care unit admission (n=10, 7.2%), pneumonia (n=9, 6.5%) and urinary tract infection (n=8, 5.8%). Overall, 3 deaths (sFI score 3+, 2 deaths; sFI score 3, 1 death) occurred during the initial 30 days after surgery (0.9% 30-day mortality rate). 90-day mortality was 2.1% (7 deaths: sFI score 3+: 3 deaths; sFI score 3: 2 deaths; sFI score 2: 2 deaths). Postoperative hospital length of stay and readmission data are summarized in **Table S1**.

**Table 2:** Description of Complications with Dindo-Clavien Grade of 2 or Greater.

Complication	No. (%)
Dindo-Clavien grade 2	95 (68.8)
Delirium	23 (16.7)
Perioperative blood transfusion	5 (3.6)
Pneumonia	9 (6.5)
Pulmonary embolism	2(1.4)
Bacteremia	1(0.7)
Urinary tract infection	8 (5.8)
Surgical site infection	6 (4.3)
Incision fat liquefaction	11 (8.0)
Ileus	3 (2.2)
Atrial fibrillation	7 (5.1)
NSTEMI	4 (2.9)
Hypocalcemia	3(2.2)
Coagulation disorder	1(0.7)
Pulmonary edema	2(1.4)
Parenteral nutrition	4(2.9)
Lymphatic fistula	6 (4.3)
Dindo-Clavien grade 3	10 (7.2)
Deep venous thrombosis	3(2.2)
Acute erosive hemorrhagic gastritis	1(0.7)
Reoperation	3(2.2)
Closed chest drainage	2(1.4)
Urine retention	1(0.7)
Dindo-Clavien grade 4	31 (22.5)
STEMI	2(1.4)
Heart failure	4 (2.9)
Vasoactive agent support	5 (3.6)
Respiratory failure	3(2.2)
Hemorrhagic shock	2(1.4)
Renal failure needing hemodialysis	2 (1.4)
Requiring intensive care unit admission	10 (7.2)
Sepsis	1(0.7)
MODS	1(0.7)
Septic shock	1(0.7)
Dindo-Clavien grade 5 (deaths)	2 (1.4)

NSTEMI, Non-ST-segment elevation myocardial infarction; STEMI, ST-segment elevation myocardial infarction; MODS, multiple organ dysfunction syndromes.

Based on VIMP and the minimal depth analyses, major predictors of postoperative complications after surgery were history of cerebral infarction, sFI score, renal failure, smoking, ASA score, age, and sex (**Supplementary Figure 1**). Moreover, we performed logistic regression analysis to determine which factors were associated with Clavien-Dindo grade ≥3 postoperative complications (**Table 3**),and found that the history of cerebral infarction, sFI, renal failure, smoking, ASA score, and age were significantly associated with postoperative complications. Multivariable analysis revealed that history of cerebral infarction (odds ratio (OR)=4.587, *p*=0.003), sFI (sFI=2~3 vs. sFI=0~1, OR=3.121, *p* = 0.031; sFI=4~5 vs. sFI=0~1, OR=6.666, *p*= 0.002), renal failure (OR=2.617, *p*=0.020), smoking (OR=2.284, *p*=0.024), and age (OR=2.174, *p*=0.035) were significant independent predictors of complications graded as Dindo-Clavien 3 or more. The C-Index of the model was 0.756 (95% confidence interval (CI),0.703-0.809).

**Table 3:** Univariate and multivariate logistic regression model assessing predictors of postoperative complications.

Variable	Univariate analysis			Multivariate analysis		
	OR	95%CI	p-value	OR	95%CI	p-value
Age						
<80	Reference			Reference		
≥80	2.671	1.407-4.988	0.002	2.174	1.050-4.465	0.035
Smoking						
No	Reference					
Yes	2.960	1.567-5.518	0.001	2.284	1.104-4.651	0.024
ASA						
<3	Reference			Reference		
≥3	3.496	1.934-6.452	<0.001	1.761	0.886-3.518	0.106
History of cerebral infarction						
No	Reference			Reference		
Yes	5.394	2.133-13.534	<0.001	4.587	1.661-12.698	0.003
sFI						
0~1	Reference			Reference		
2~3	5.289	2.175-15.839	<0.001	3.121	1.190-9.775	0.031
4~5	16.008	5.729-52.663	<0.001	6.666	2.127-23.747	0.002
Renal failure						
No	Reference			Reference		
Yes	3.100	1.506-6.208	0.002	2.617	1.146-5.838	0.020
Sex						
Female	Reference					
Male	0.563	0.311-1.006	0.053			

Based on the results of multivariable analysis, five factors were combined to establish a nomogram (**Supplementary Figure 2**). AUC analysis of ROC curves was used to evaluate the discrimination performance of the nomogram. As shown in **Supplementary Figure 3**, the AUC value, calibration plot, and DCA curves for the model suggested acceptable performance and discrimination, calibrating ability, and clinical usefulness.

### 3.3 Overall survival and cancer-specific survival according to sFI

**Figure 1:**
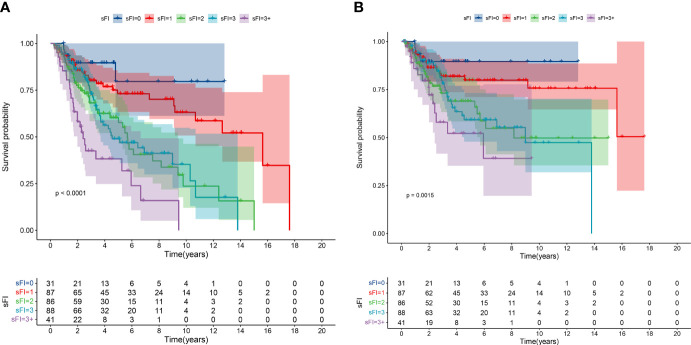
Kaplan–Meier curves for overall survival (OS) **(A)** and cancer-specific survival (CSS) **(B)** in patients with UTUC according to sFI.

**Figure 2:**
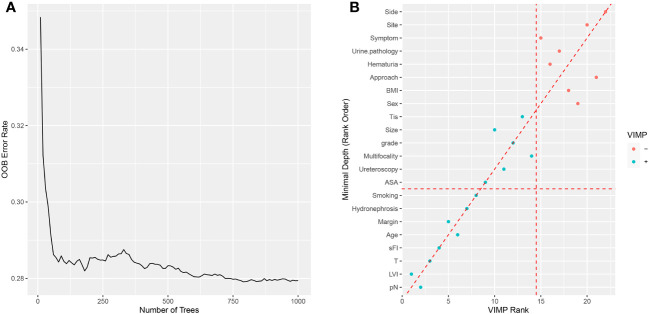
Random survival forest for OS. **(A)** The prediction error rate for random survival forests of 1000 trees. **(B)** Variables selected by VIMP and minimal depth.

As shown in **Figure 1**, we analyzed OS and CSS curves according to sFI. There were significant survival differences in OS and CSS (*p*<0.001) among the five groups of patients classified by sFI. Eight variables selected by VIMP and minimal depth methods were chosen for subsequent modeling (variables below the horizontal dotted line) (**Figure 2**). Based on univariate analysis, age, smoking, hydronephrosis, LVI, T stage, N stage, margin, and sFI score were significantly associated with worse survival rate. Those eight variables were included in multivariate Cox regression analysis, which showed that age (hazard ratio (HR)=1.055, *p*=0.003), hydronephrosis (HR=1.779, *p*=0.019), LVI (HR=2.087, *p*=0.003), T stage (T2 vs. T1, HR=1.798, *p* = 0.037; T3 vs. T1, HR=2.219, *p* = 0.003; T4 vs. T1, HR=3.476, *p* = 0.002), N stage (HR=3.170, *p*<0.001), margin (HR=2.517, *p*=0.015), and sFI score (sFI=2 vs. sFI=0, HR=3.025, *p* = 0.037; sFI=3 vs. sFI=0, HR=3.407, *p* = 0.023; sFI=3+ vs. sFI=0, HR=3.965, *p* = 0.016) were all significant prognostic factors for OS (**Table 4**).

**Table 4:** Univariate and Multivariate Cox Analyses for OS of UTUC Patients.

Variable	Univariate analysis			Multivariate analysis		
	HR	95%CI	p-value	HR	95%CI	p-value
Age						
<80	Reference			Reference		
≥80	1.064	1.033-1.096	<0.001	1.055	1.018-1.093	0.003
Smoking						
No	Reference					
Yes	1.607	1.082-2.385	0.019	1.313	0.842-2.047	0.230
Hydronephrosis						
No	Reference			Reference		
Yes	2.211	1.398-3.498	<0.001	1.779	1.099-2.879	0.019
LVI						
No	Reference			Reference		
Yes	3.616	2.438-5.364	<0.001	2.087	1.285-3.391	0.003
T stage						
T1	Reference			Reference		
T2	1.908	1.120-3.251	0.017	1.798	1.036-3.123	0.037
T3	2.512	1.517-4.159	<0.001	2.219	1.301-3.786	0.003
T4	9.731	5.005-18.920	<0.001	3.476	1.541-7.838	0.002
Margin						
Negative	Reference			Reference		
Positive	4.412	2.292-8.492	<0.001	2.517	1.195-5.303	0.015
N stage						
pN0&Nx	Reference			Reference		
pN+	5.146	3.205-8.264	<0.001	3.170	1.770-5.676	<0.001
sFI						
0	Reference			Reference		
1	1.698	0.590-4.891	0.326	1.368	0.469-3.989	0.566
2	4.147	1.485-11.579	0.007	3.025	1.067-8.576	0.037
3	3.916	1.402-10.934	0.009	3.407	1.182-9.821	0.023
3+	8.437	2.939-24.222	<0.001	3.965	1.296-12.132	0.016

LVI, lympho-vascular invasion; sFI, simplified five-item frailty index.

Regarding CSS, seven variables were included in the univariate analysis (**Supplementary Figure 4**; **Table 5**). Six factors(hydronephrosis, LVI, T stage, N stage, margin, and sFI score)had significant effects on CSS and were identified as prognostic factors (*p* < 0.05) (**Table 5**).

**Table 5:** Univariate and Multivariate Cox Analyses for CSS of UTUC Patients.

Variable	Univariate analysis			Multivariate analysis		
	HR	95%CI	p-value	HR	95%CI	p-value
Smoking						
No	Reference					
Yes	1.800	1.116-2.905	0.016	1.574	0.943-2.628	0.083
Hydronephrosis						
No	Reference			Reference		
Yes	2.461	1.336-4.534	0.004	2.019	1.071-3.806	0.030
LVI						
No	Reference			Reference		
Yes	3.737	2.311-6.042	<0.001	2.165	1.220-3.843	0.008
T stage						
T1	Reference			Reference		
T2	1.852	0.867-3.959	0.112	1.909	0.875-4.167	0.104
T3	3.873	1.960-7.655	<0.001	3.328	1.625-6.817	0.001
T4	11.689	4.824-28.325	<0.001	4.933	1.750-13.905	0.003
Margin						
Negative	Reference			Reference		
Positive	4.501	2.059-9.839	<0.001	3.337	1.431-7.779	0.005
N stage						
pN0&Nx	Reference			Reference		
pN+	6.046	3.436-10.640	<0.001	3.225	1.733-6.004	<0.001
sFI						
0	Reference			Reference		
1	1.642	0.478-5.647	0.431	1.174	0.336-4.107	0.802
2	3.566	1.079-11.787	0.037	2.493	0.743-8.363	0.139
3	3.504	1.067-11.508	0.038	3.462	1.040-11.525	0.043
3+	5.659	1.623-19.729	0.007	3.831	1.065-13.776	0.040

LVI, lympho-vascular invasion; sFI, simplified five-item frailty index.

### 3.4 Development and validation of a novel prognostic nomogram

**Figure 3:**
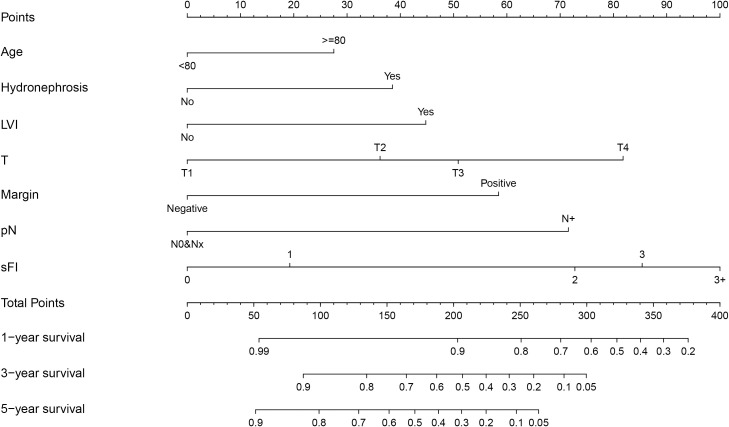
Establishment of overall survival (OS) nomogram.

**Figure 4:**
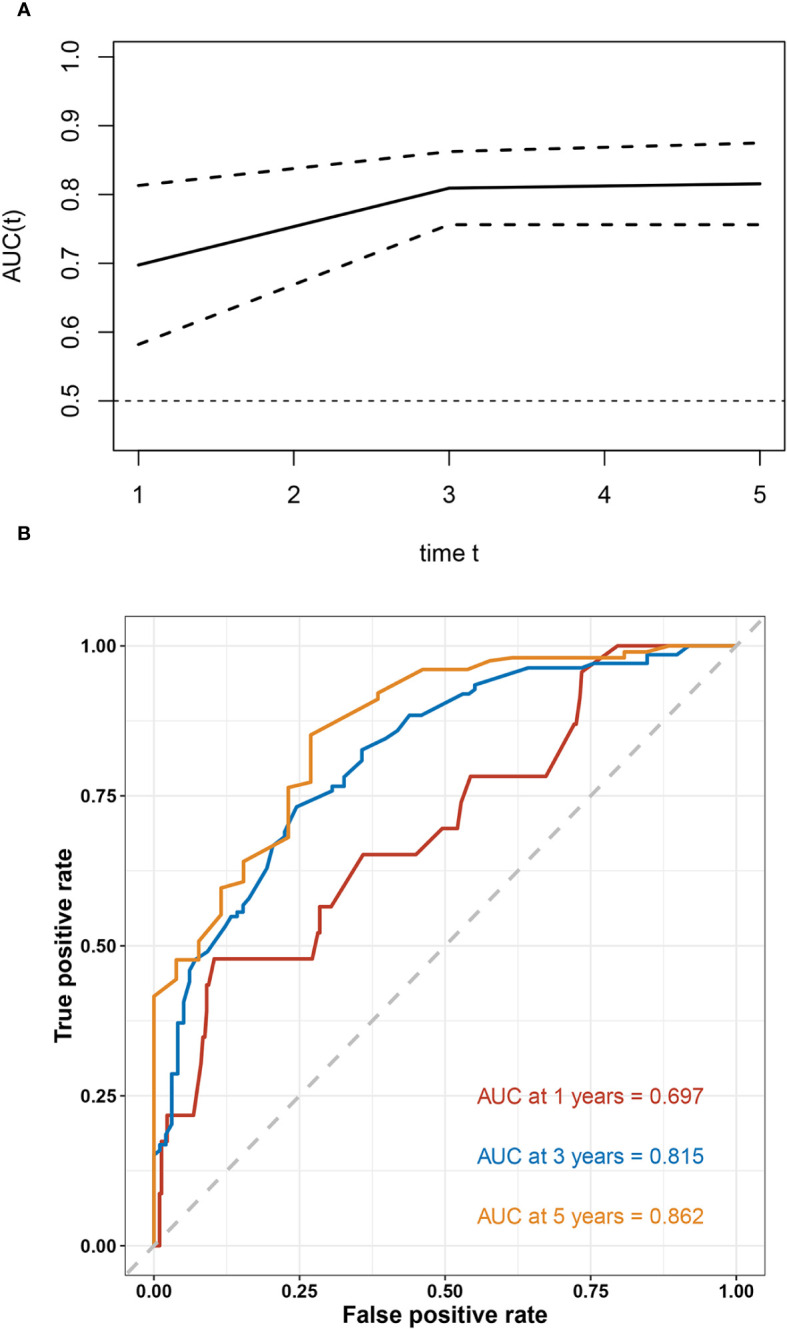
Evaluation of the discriminative ability of the OS nomogram. The time-independent AUC **(A)** and ROC curves **(B)** for the nomogram.

**Figure 5:**
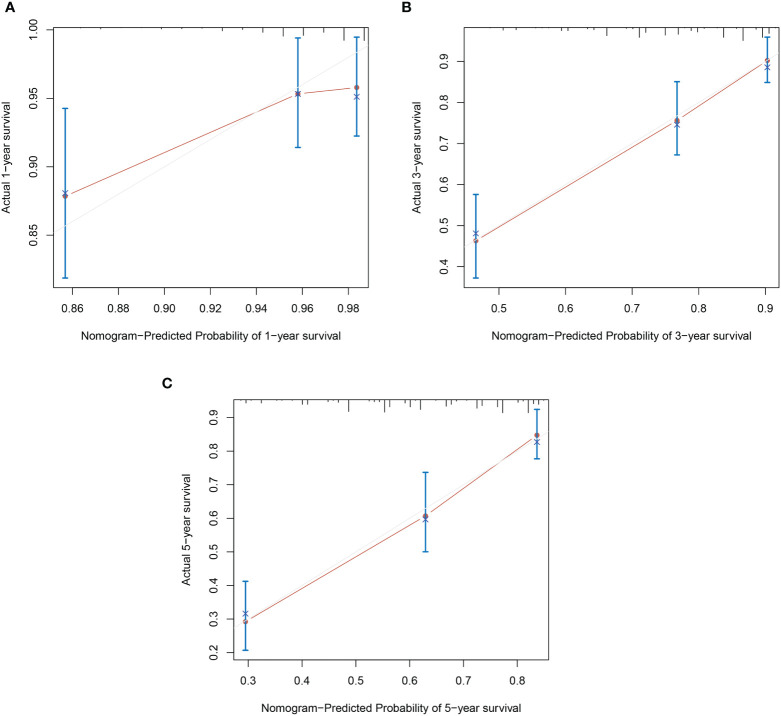
Calibration plots of OS nomogram model. **(A)** 1-year calibration plot of OS; **(B)** 3-year calibration plot of OS; **(C)** 5-year calibration plot of OS.

**Figure 6:**
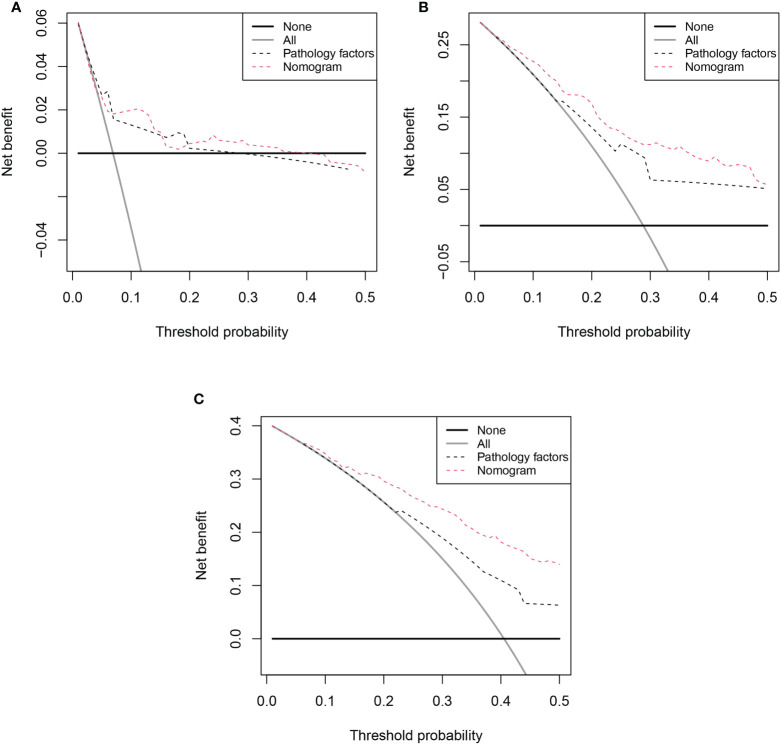
Decision curve analysis (DCA) of OS nomogram. **(A)** 1-year DCA of nomogram; **(B)** 3-year DCA of nomogram; **(C)** 5-year DCA of nomogram;.

Based on the results of multivariable analysis, a nomogram for OS and CSS prediction was constructed (**Figure 3**; **Supplementary Figure 5**). C-index values of the nomogram for OS and CSS prediction were 0.750 (95% CI, 0.704–0.796) and 0.781 (95% CI, 0.732–0.830), respectively. In addition, the time-dependent AUC values for predicting OS and CSS within 5 years were both > 0.7 for predicting OS and CSS within 5 years (**Figure 4**; **Supplementary Figure 6**), indicating good discrimination by the nomogram. The 1-, 3-, and 5-year AUC values for OS were 0.697, 0.815, and 0.862, respectively (**Figure 4**), while for CSS, the 1-, 3-, and 5-year AUC values were 0.772, 0.823, and 0.843, respectively (**Supplementary Figure 6**). Calibration curves showed good agreement between the predicted and observed risks for both OS and CSS (**Figure 5**; **Supplementary Figure 7**). As shown in **Figure 6** and **Supplementary Figure 8**, DCA suggested that the nomogram demonstrated a higher net benefit than pathology factors for predicting 1-, 3-, and 5-year OS and CSS.

### 3.5 Risk stratification based on the nomogram

**Figure 7:**
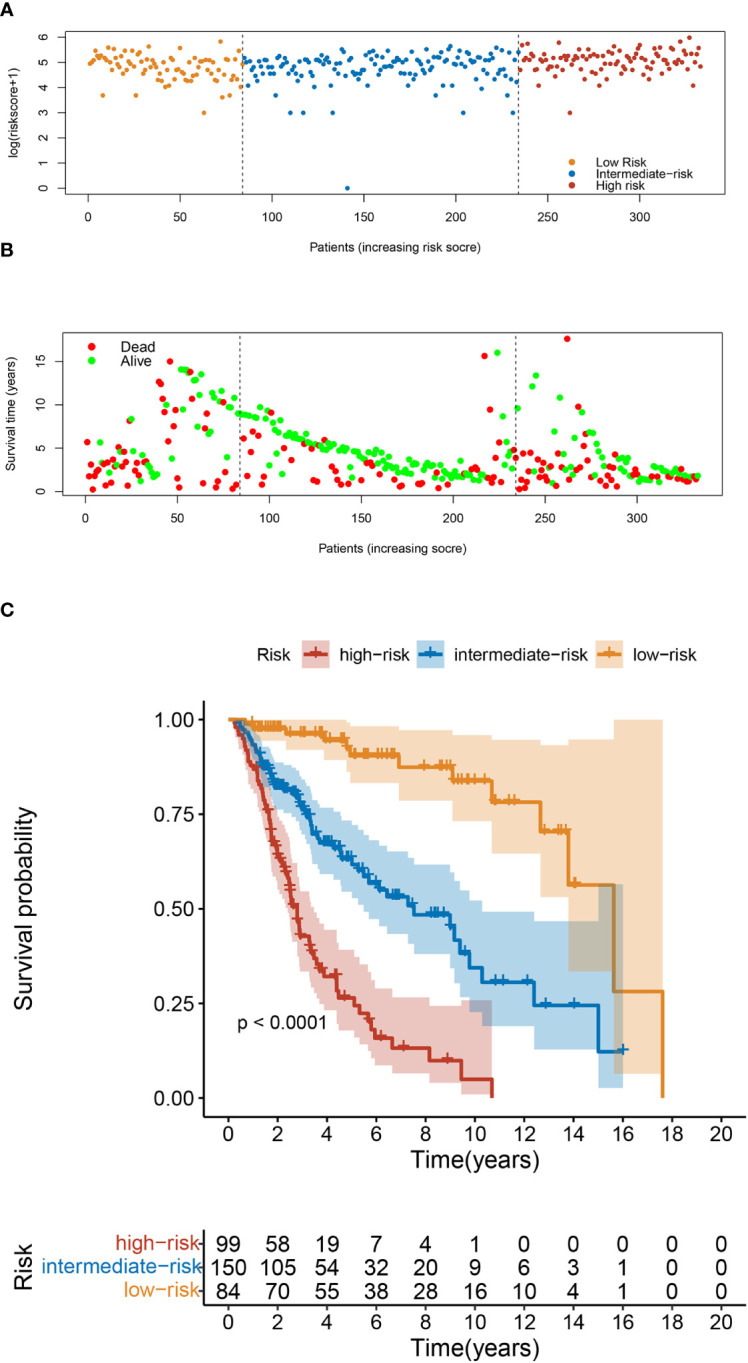
Risk Classification based on OS nomogram. **(A)** Survival status map. **(B)** Risk heatmap. **(C)** Kaplan–Meier overall survival curves of patients with UTUC with different risks stratified by the nomogram.

Finally, we constructed a risk classification systembased on the patient total nomogram scores. Patients were divided into three groups: low-risk (score < 95), intermediate-risk (95 ≤ score < 175), and high-risk (score ≥ 175). Significant differences were detected among the Kaplan-Meier curves for OS and CSS of the three groups (**Figure 7**; **Supplementary Figure 9**).

## 4 Discussion

Among all types of cancer, upper tract urothelial carcinoma is one of the most aggressive. RNU with bladder cuff resection is the gold-standard treatment for high-risk UTUC. However, surgery has considerable risks, despite its excellent oncological outcomes. It was reported that 20.5% of patients experienced morbidity, and 1.5% died within 30 days after surgery (34). Moreover, the growing geriatric population and advance in minimally invasive procedures have boosted the demand for radical procedures, such as RNU, in the elderly. But a lower physiological reserve mean that risks are higher for older adults. Therefore, it is very important to assess the risk of complications and prognosis in the elderly. It was reported recently that frailty is strongly associated with postoperative morbidity and in-hospital mortality in older patients (20, 35); however, the association between frailty and outcomes in UTUC is unknown.

To our best knowledge, this was the first study to validate the effectiveness of the sFI in older patients with UTUC undergoing RNU. One strength and a novelty feature of the present study is the application of machine learning methods. By employing a random forest model, we determined the importance of each variable using the VIMP and minimal depth methods. We found that sFI was associated with postoperative complications, consistent with previous studies. Further, we constructed a nomogram based on five variables with a good predictive value for postoperative complications in elderly patients with UTUC. Moreover, we assessed the relationship between sFI and prognosis, and found that high sFI scores were significantly associated with worse OS (*p*<0.001) and CSS(*p*=0.0015). High sFI score was identified as an independent risk factor for OS and CSS. We also integrated sFI and clinical variables into a nomogram to predict OS and CSS, where nomograms have shown excellent ability to individualize risk stratification.

Frailty is characterized by multifactorial deterioration in energy metabolism, strength, endurance, and function beyond what is expected for a person of a given age (6, 7, 36, 37). Meta-analysis showed that the prevalence of frailty among older adults undergoing general surgery ranged from 8% to 77.8% (5). Recently, frailty has been increasingly recognized as a common feature affecting surgical outcomes, such as increased risk of postoperative complications, mortality, and prolonged hospital stay. The mFI-11 index is used to evaluate frailty and predict postoperative mortality and morbidity (18, 38); for example, Dicpinigaitis and colleagues reported that higher mFI-11 score is associated with development of severe complications, but not with in-hospital mortality or extended length of stay (39). In addition, Heimann et al. found that preoperative frailty independently predicts OS in elderly patients with brain metastases requiring surgery (40). Banaszek et al. also concluded that mFI-11 is an independent predictor of adverse events, acute length of stay, and in-hospital mortality in patients after traumatic spinal cord injury (41); however, there remains a lack of consensus regarding the best way to assess frailty, in terms of feasibility and reliability in daily clinical practice.

Consequently, the reorganized 5-item modified frailty index (mFI-5) was developed based on five routinely collected patient comorbidity factors. According to the present study, patients with higher mFI-5 score had higher levels of perioperative complication and worse survival rates, consistent with previous findings (9). Additionally, Subramaniam et al. found that the mFI-5 was a significant independent predictor of mortality and postoperative complications (42). Likewise, in a retrospective National Surgical Quality Improvement Program study of 336,556 patients undergoing primary hip and knee arthroplasty, Traven et al. found that frailty was a significant independent predictor of postoperative complications, including life-threatening medical complications, surgical site infections, readmission, and mortality within 30 days (21). Similarly, in a multicenter retrospective study, Yamashita et al. showed that high preoperative modified 5-item frailty index score was a significant independent predictor of poor OS (23). These studies support our conclusion that the 5-item modified frailty index (mFI) is a proven and reliable method to characterize frailty and as associated with postoperative complications and poor prognosis. Therefore, surgical and prognostic risk stratification based on mFI-5 score can help surgeons assess surgical risk and predict patient prognosis, as well as guiding post-surgery recovery plans and assisting in the prevention of complications in high-risk patients.

Our study indicated that sFI has significant impact on CSS. However, Other studies suggest that frailty is associated with perioperative and short-term outcomes but not cancer-specific outcomes (43). This may be attributed to various factors. Firstly, these studies assessed frailty differently, which may lead to heterogeneity. Further, the follow-up period is short, which may have affected survival outcomes. Secondly, elevated levels of some inflammatory cytokines have been reported in frail patients (44), suggesting that chronic inflammation may play a role. Chronic inflammation is closely related to the occurrence and progression of tumors. In addition, frail patients cannot tolerate the trauma and side effects of treatment, which will also affect the patient’s prognosis. Although the underlying mechanisms between frailty and poor prognosis are not well understood, these reasons may explain the poorer prognosis of frail patients. We also found that smoking was a risk factor for major complications but not for long-term outcomes. Many previous studies have shown that smoking is a risk factor for postoperative complications (45, 46). However, there have been few studies on the relationship between smoking and postoperative complications of UTUC. Therefore, our results can provide guidance for the perioperative period, and indicate that smoking cessation is necessary for patients undergoing surgery for UTUC. However, in terms of long-term prognosis, smoking is not associated with non-negligible differences in overall mortality and cancer specific mortality rates. This finding is inconsistent with those of previous studies (46, 47), possibly because the population included in our study was older, potentially resulting in selection bias. Further, our follow-up period was short, which may have affected survival outcomes.

This study had several limitations. First, selection bias may have occurred because the study was conducted in a single center. Our data show that the prevalence of frailty was 90%; the main reason for this is that our hospital is a national geriatric center, and most patients admitted to the hospital have multiple comorbidities, leading to a greater proportion of patients with high frailty scores. Second, although we internally validated the predictive value of sFI, our findings were not externally validated using an independent dataset. In addition, this study is limited by the limitations of the recording variables. Finally, physical and environmental factors can affect the prognosis of elderly patients with cancer. Therefore, it is essential to account for additional confounding factors that may influence patient outcomes.

## 5 Conclusion

Overall, our data demonstrate an association between frailty and RNU outcomes. sFI represents a potential predictor of procedure-related complications and prognosis in older patients with UTUC patients. Moreover, the nomogram developed in this study incorporates sFI and clinical risk factors to effectively predict OS and CSS in elderly patients with UTUC following RNU.

## Data availability statement

The original contributions presented in the study are included in the article/**Supplementary Material**. Further inquiries can be directed to the corresponding authors.

## Author contributions

JYL: Project development, Data collection, Data analysis, Manuscript writing. HRW: Project development, Data collection, Data analysis, Manuscript writing. PJW: Data collection, Data analysis, Manuscript editing. JWW: Data collection, Data analysis. JYW: Data analysis, Manuscript editing. HMH: Project development, Data analysis, Manuscript editing. JLW: Project development, Data analysis, Manuscript editing. YGZ: Project development, Data analysis, Manuscript editing. All authors contributed to the article and approved the submitted version.

## Supplementary material

Supplementary Table 1 Length of Stay and readmission by groups of sFIFigure captions.
