# Corrigendum: A simplified frailty index and nomogram to predict the postoperative complications and survival in older patients with upper urinary tract urothelial carcinoma

**DOI:** 10.3389/fonc.2025.1575599

**Published:** 2025-03-04

**Authors:** Jianyong Liu, Haoran Wang, Pengjie Wu, Jiawen Wang, Jianye Wang, Huimin Hou, Jianlong Wang, Yaoguang Zhang

**Affiliations:** ^1^ Beijing Hospital, National Center of Gerontology, Institute of the Geriatric Medicine, Chinese Academy of Medical Sciences & Peking Union Medical College, Beijing, China; ^2^ Beijing Hospital Continence Center, Beijing, China

**Keywords:** upper urinary tract urothelial carcinoma, frailty, postoperative complication, prognosis, risk stratification, random forest

In the published article, there was an error in affiliations 1 and 2. Instead of affiliations 1 “Department of Urology, Beijing Hospital, National Center of Gerontology, Institute of the Geriatric Medicine, Chinese Academy of Medical Sciences, Beijing, China”, affiliation 2 ^“^Graduate School of Peking Union Medical College and Chinese Academy of Medical Sciences, Beijing, China” and affiliation 3 “Beijing Hospital Continence Center, Beijing, China”, it should be affiliation 1 “Beijing Hospital, National Center of Gerontology, Institute of the Geriatric Medicine, Chinese Academy of Medical Sciences & Peking Union Medical College, Beijing, China” and affiliation 2 “Beijing Hospital Continence Center, Beijing, China”.

The authors apologize for this error and state that this does not change the scientific conclusions of the article in any way. The original article has been updated.

