# Supplementary material for: A simplified frailty index and nomogram to predict the postoperative complications and survival in older patients with upper urinary tract urothelial carcinoma
Source: Front Oncol. 2023 Oct 11;13:1187677. doi: 10.3389/fonc.2023.1187677 (PMC10600399; doi:10.3389/fonc.2023.1187677)
Supplement: Supplementary Table 1 — Length of Stay and readmission by groups of sFIFigure captions. [file DataSheet_1.docx]

Supplementary Material

**Title**

**A simplified frailty index and nomogram to predict the postoperative complications and survival in older patients with upper urinary tract urothelial carcinoma**

Jianyong Liu^1,2,3^†, Haoran Wang^1,2,3^†, Pengjie Wu^1,3^, Jiawen Wang^1,2,3^, Jianye Wang^1,2,3^, Huimin Hou^1,2,3*^, Jianlong Wang^1,2,3*^, Yaoguang Zhang^1,2,3*^

**Corresponding Author:**

Huimin Hou，

address:No. 1 DaHua Road, Dong Dan, Beijing, 100730, China.

Email: houhuimin0305@163.com, phone: +8618610512795, fax: +86 10 85136272,

Jianlong Wang,

address:No. 1 DaHua Road, Dong Dan, Beijing, 100730, China.

Email: wjlspplaaa@sina.com, phone: +8613522047356, fax: +86 10 85136272

Yaoguang Zhang,

address: No. 1 DaHua Road, Dong Dan, Beijing, 100730, China.

Email: zhangyaoguang3247@bjhmoh.cn, phone: +8613031099662, fax: +86 10 85136272.

# Supplementary Table

**Table S1. Length of Stay and readmission by groups of sFI**

|  | **0** | **1** | **2** | **3** | **3+** | **p-value** |
| --- | --- | --- | --- | --- | --- | --- |
|  | **N=31** | **N=87** | **N=86** | **N=88** | **N=41** |  |
| Readmission (number) (%) | 2 (5.1) | 4 (10.3) | 7 (17.9) | 11 (28.2) | 15 (38.5) | 0.220 |
| Length of stay (days) (Mean ±SD) | 9.48±2.6 | 10.8±3.9 | 11.5±6.3 | 12.0±7.3 | 12.5±7.1 | 0.114 |

# Supplementary Figures

**
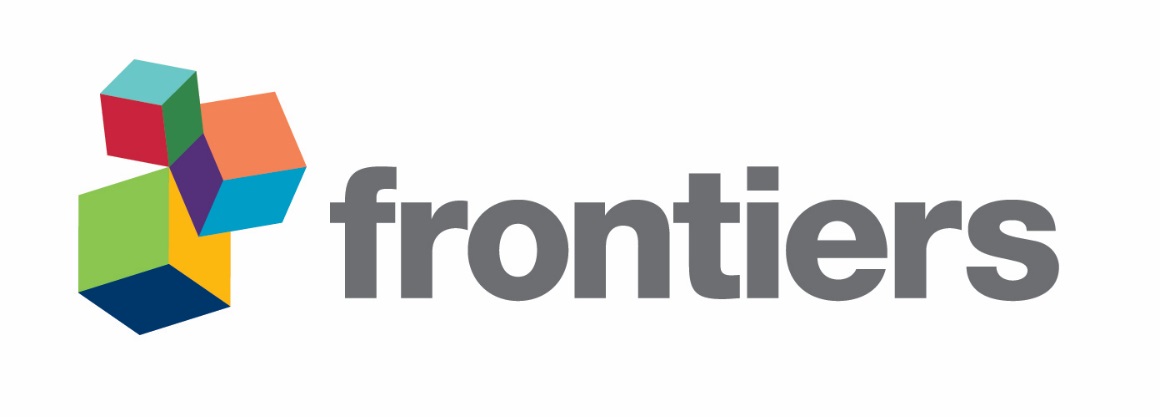
**


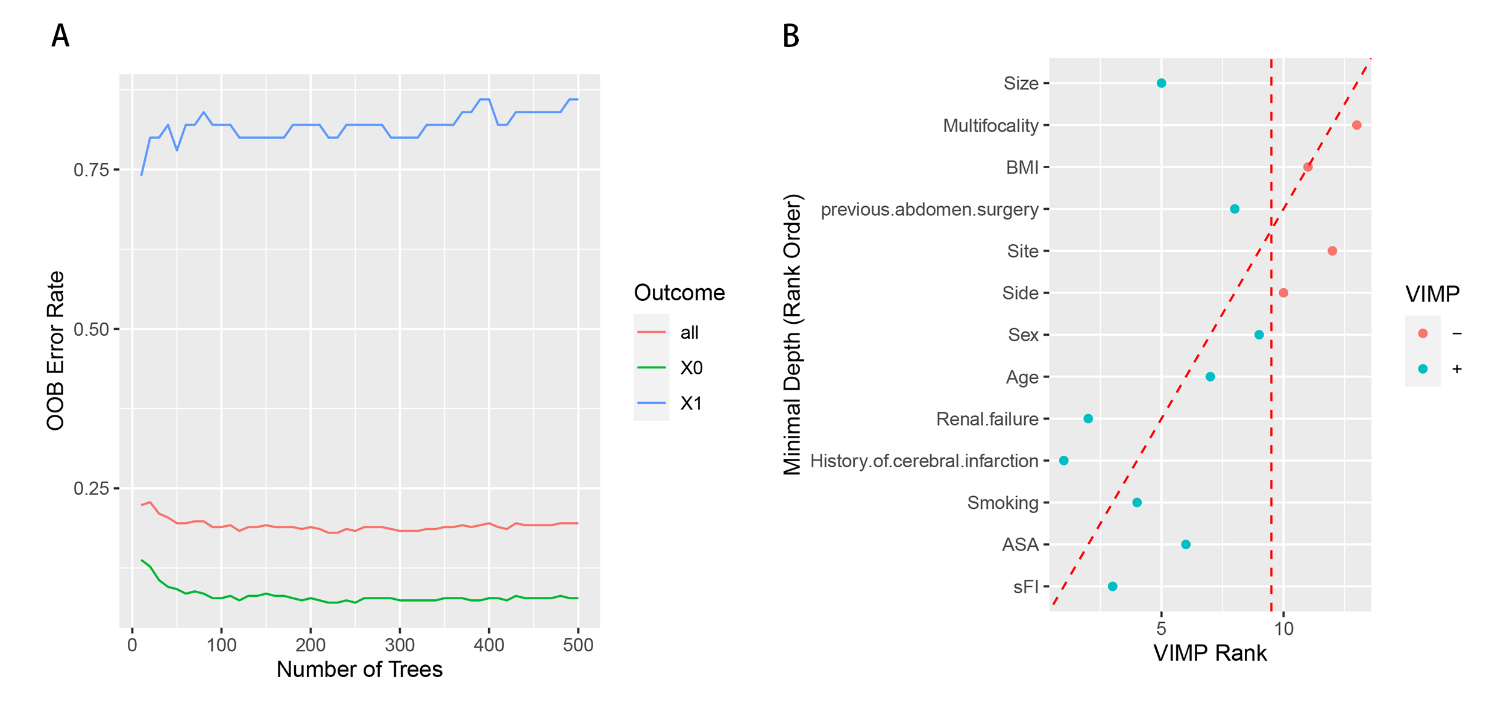
**Supplementary Figure 1** Random forest. **(A) The prediction error rate for random forests of 1000 trees. (B)** Variables selected by VIMP and minimal depth.





**Supplementary Figure 2** Establishment of postoperative complications nomogram.


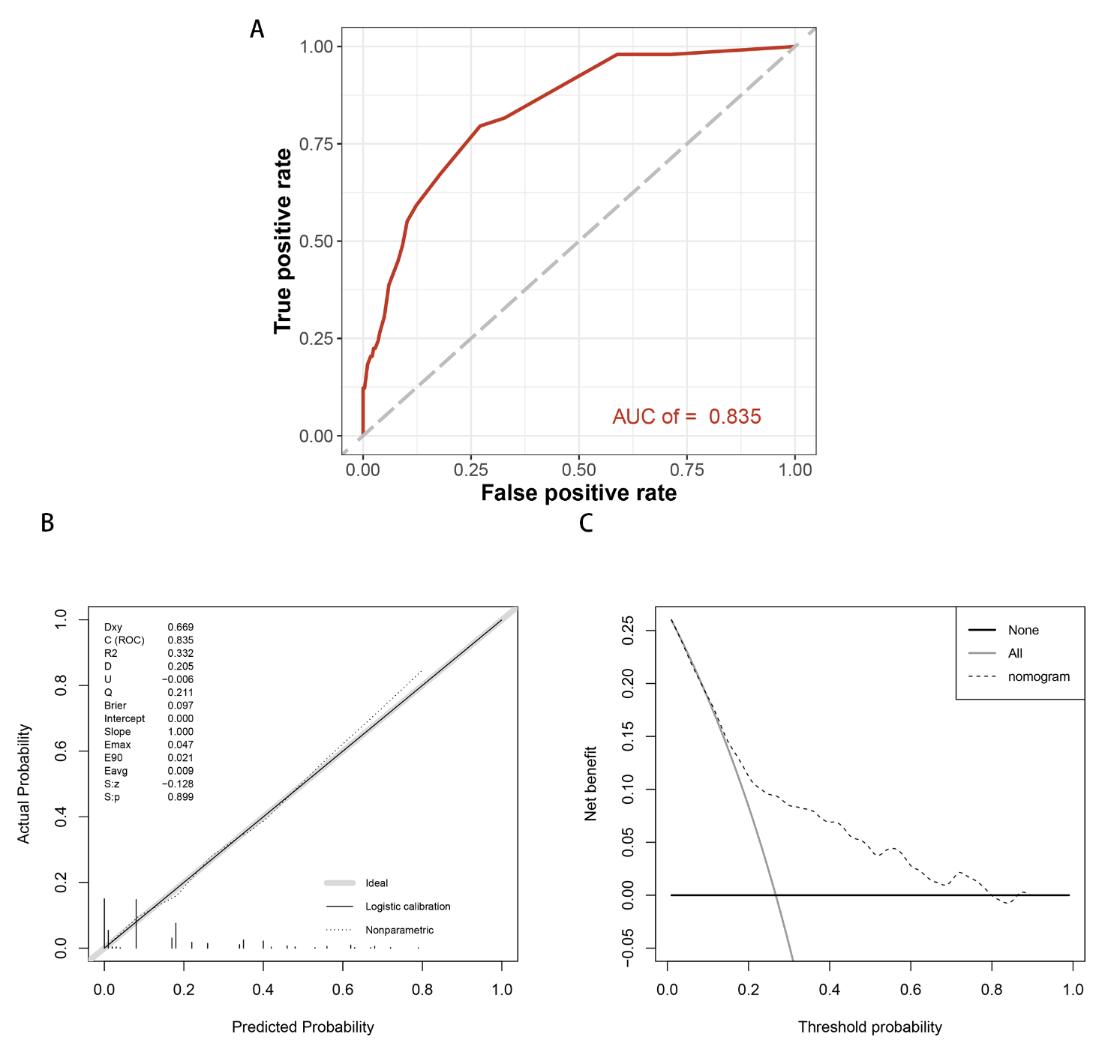


**Supplementary Figure 3** The evaluation and validation of the nomogram. (**A**) Receiver operating characteristic curve for the nomogram model. (**B**) Calibration curves for the nomogram. (**C**) Decision curve analysis for the nomogram.


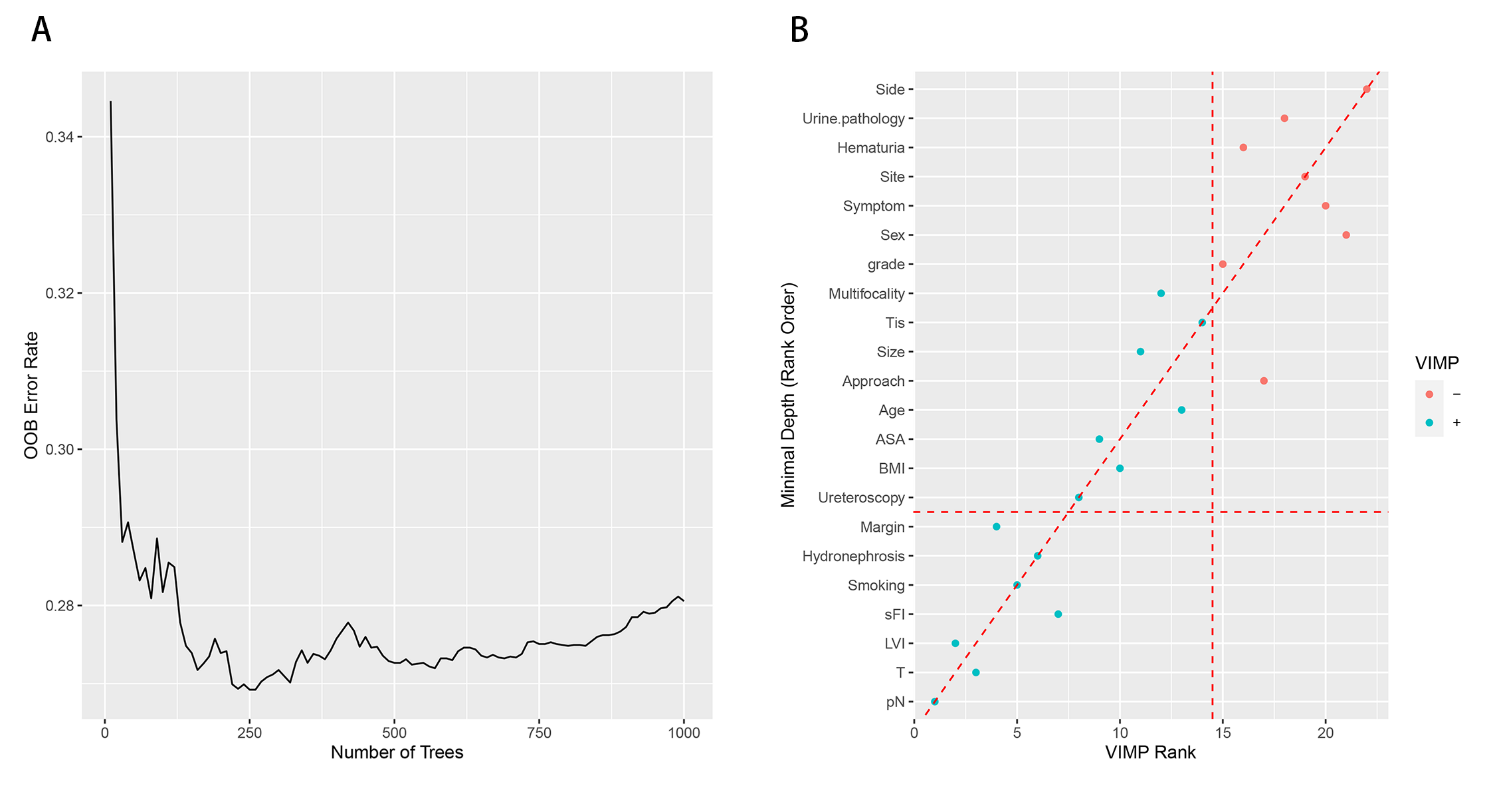


**Supplementary Figure 4** Random survival forest for CSS. **(A)** **The prediction error rate for random survival forests of 1000 trees. (B)**Comparing minimal depth and variable importance (VIMP) rankings.


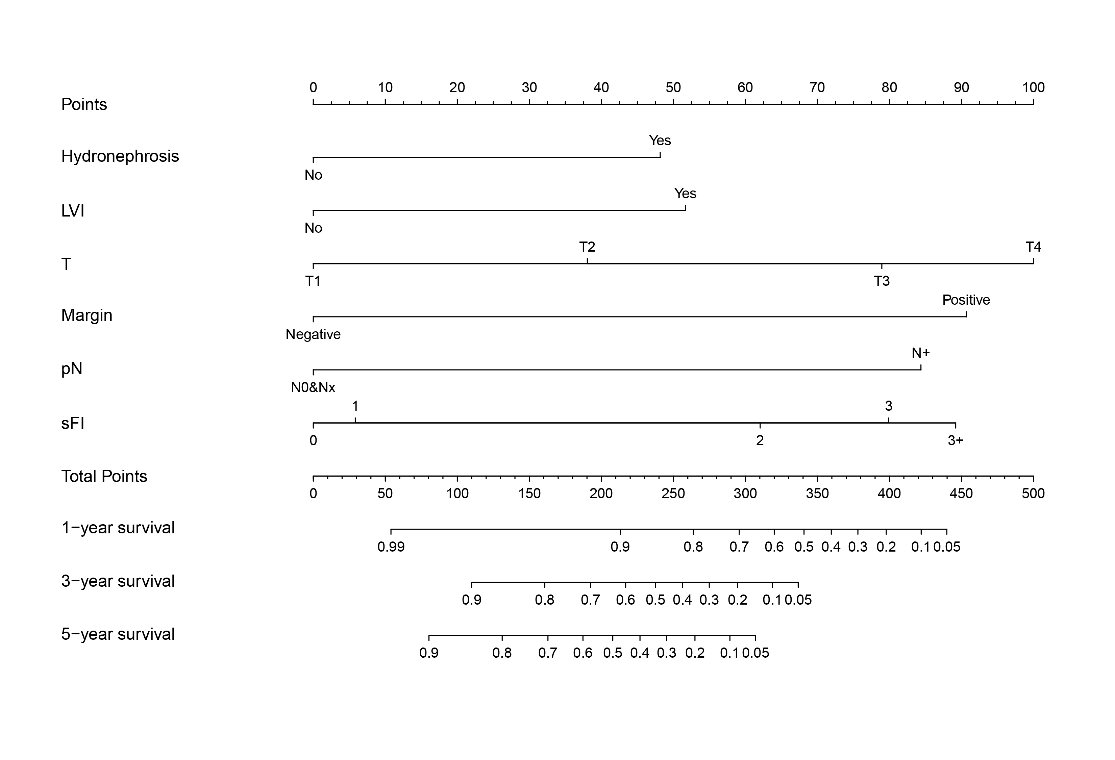


**Supplementary Figure 5** Establishment of cancer-specific survival (CSS) nomogram.


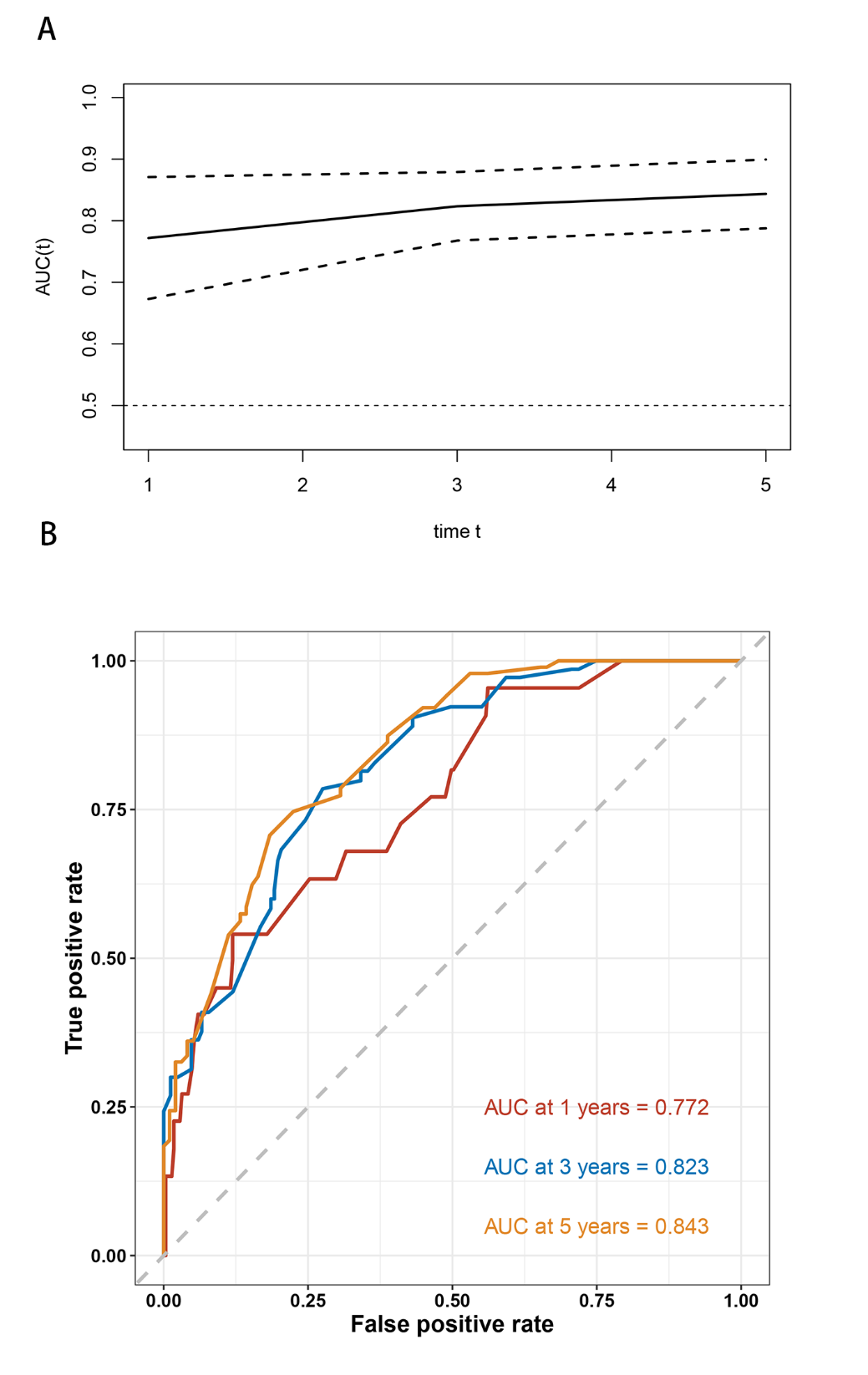


**Supplementary Figure 6** Evaluation of the discriminative ability of the CSS nomogram. The time-independent AUC **(A)** and ROC curves **(B)** for the nomogram.


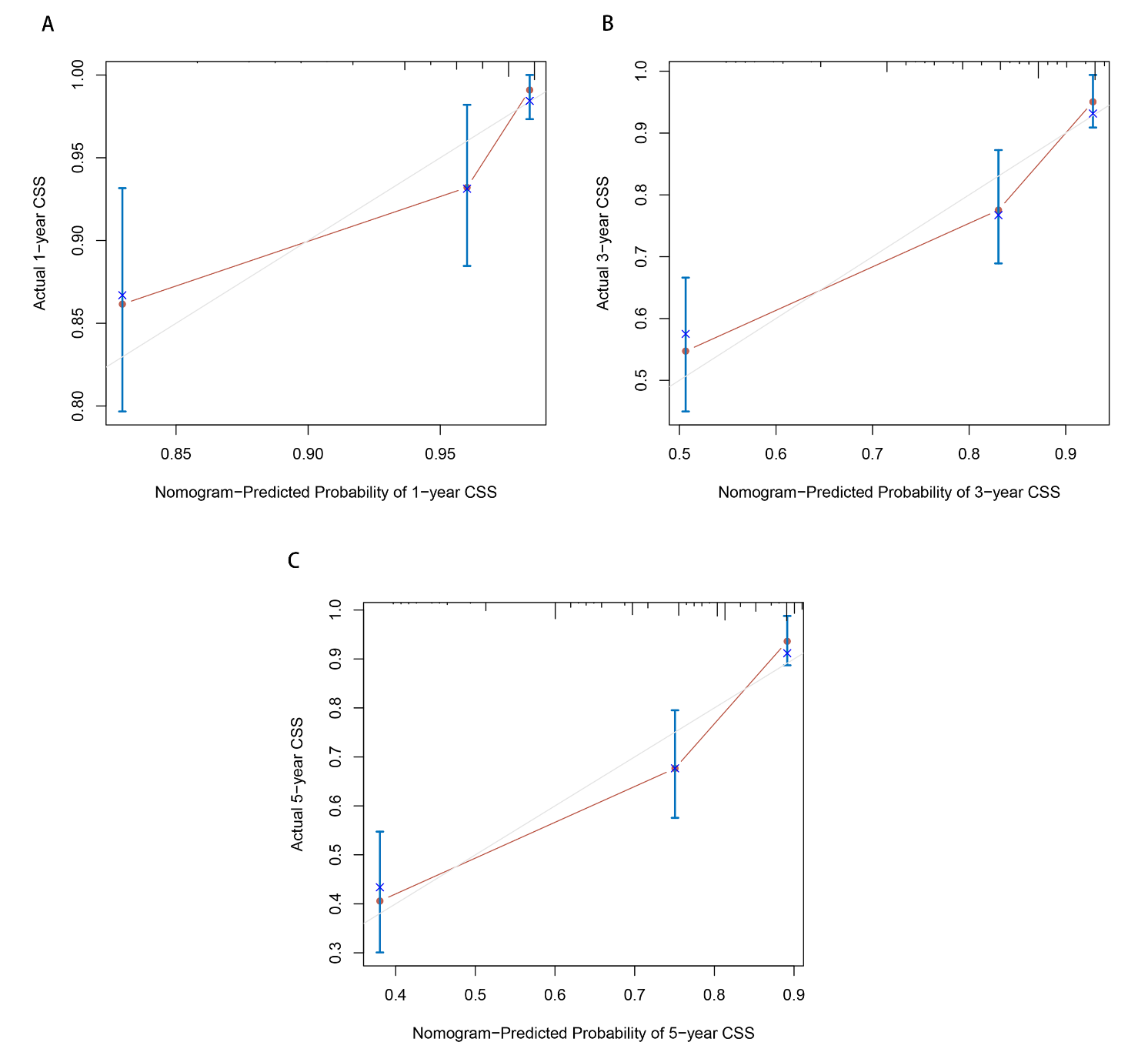


**Supplementary Figure 7** Calibration plots of CSS nomogram model. **(A)** 1-year calibration plot of CSS; **(B)** 3-year calibration plot of CSS; **(C)** 5-year calibration plot of CSS.


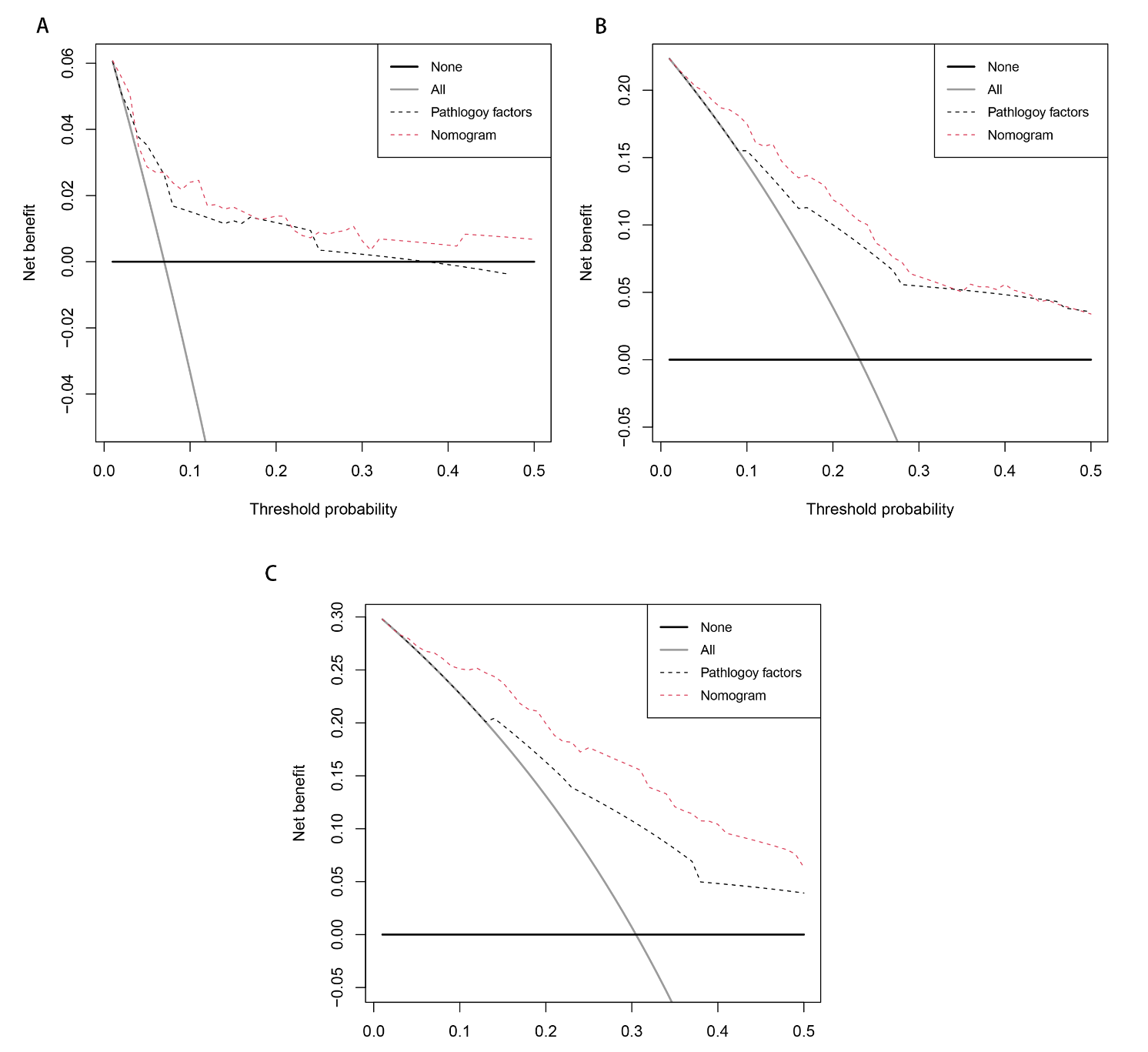


**Supplementary Figure 8** Decision curve analysis (DCA) of CSS nomogram. **(A)** 1-year DCA of nomogram; **(B)** 3-year DCA of nomogram; **(C)** 5-year DCA of nomogram;


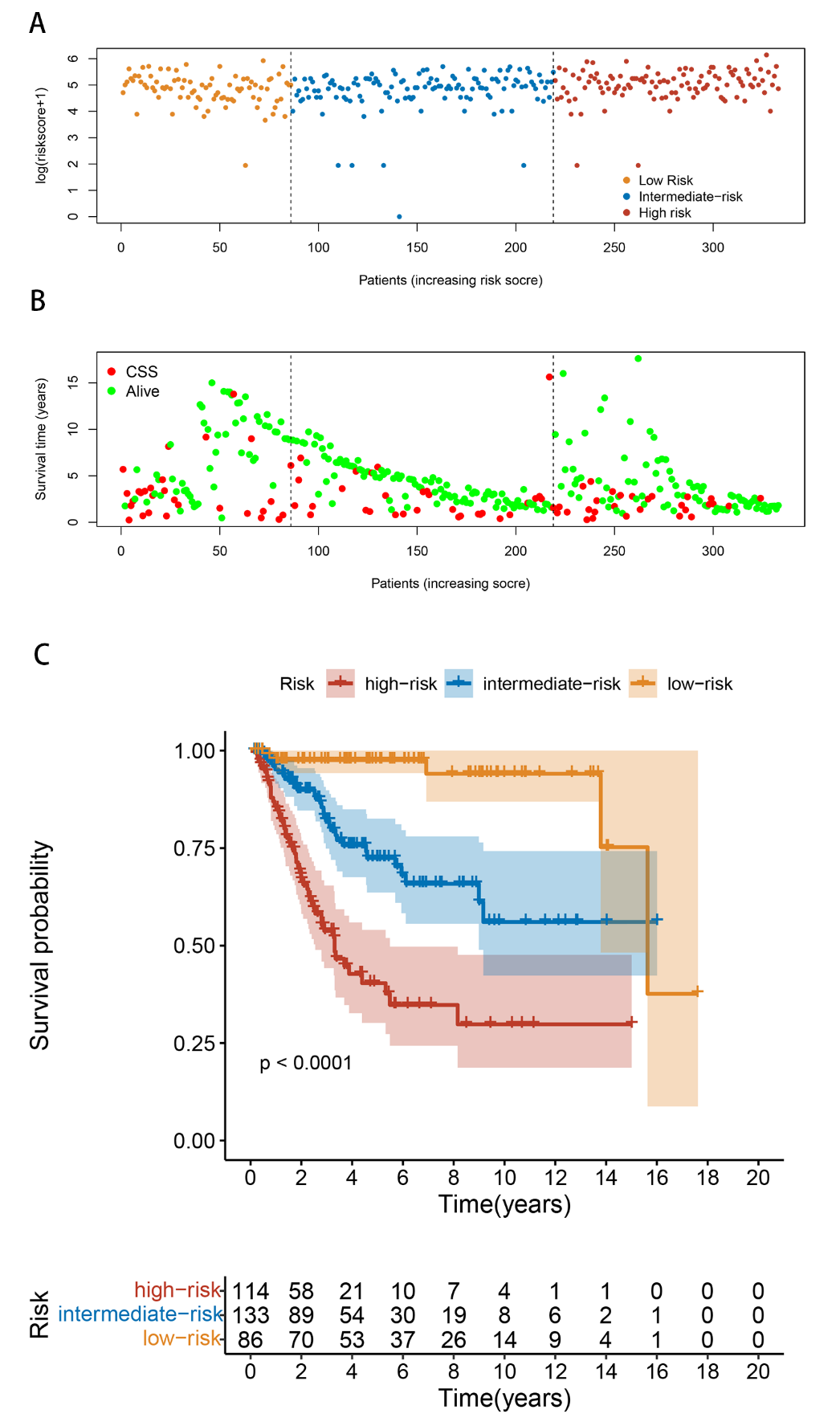


**Supplementary Figure 9** Risk Classification based on CSS nomogram. **(A)** Survival status map. **(B)** Risk heatmap. **(C)** Kaplan–Meier CSS curves of patients with UTUC with different risks stratified by the nomogram.
